# Effective Use of Topical Sucralfate in the Conservative Management of Expanded Gastrostomy Tract Reduction

**DOI:** 10.1097/PG9.0000000000000111

**Published:** 2021-07-22

**Authors:** Hengqi Betty Zheng, Mary Len, Nicole Pattamanuch

**Affiliations:** From the Division of Pediatric Gastroenterology and Hepatology, University of Washington, Seattle Children’s Hospital, Seattle, WA.

**Keywords:** gastrostomy site, peristomal healing, sucralfate

## Abstract

Sucralfate is a common medication used to treat duodenal ulcers, gastric ulcers, and gastritis. The off-label use of topical sucralfate has been described in the literature to induce wound healing in epithelial injury. Yet, current literature lacks clinical depictions in the application of sucralfate to treat a common gastrostomy tube complication, that of a dilated gastrostomy site. We present a case report of a medically complex pediatric patient where topical sucralfate was applied to reduce the size of a large gastrostomy stomal defect. Sucralfate was used to reduce healing time and allow introduction of a new gastrostomy device through the same stomal opening without the need for additional procedures or surgeries.

## INTRODUCTION

Gastrostomy tubes (G-tubes) are commonly used in pediatric patients to aid in the delivery of enteral nutrition and medication. Depending on the underlying medical condition, patients may require chronic G-tube access or short intermittent application in the setting of acute illness. Longer-term G-tube utilization can predispose to complications such as inadvertent removal/misplacement, tube malfunction, ostomy site issues such as cellulitis, discharge and leakage, peristomal abscess, bleeding, granulation tissue, and enlarged stoma site (1). Large stomal defects and subsequent increased drainage leading to persistent gastrocutaneous fistulae can present a distressing problem for gastroenterologists, patients, and caregivers. Highly acid gastric contents leaking through the gastrostomy contribute to tract inflammation and breakdown, leading to expanded stomal size and resistance to healing. Leakage also leads to skin breakdown around the stoma. Persistent gastrocutaneous fistulae have been shown to affect 30%–45% of patients with associated risk factors of young age (<2 y of age) at placement, longer duration of use, site infection, upsizing G-tube, changing from percutaneous endoscopic gastrostomy to button, and associated Nissen fundoplication procedures (2–4). Often, surgical or endoscopic closure is needed for large stomal defects and persistent tracts, incurring the need for replacement of G-tube as a secondary procedure after a lengthy period of healing.

Sucralfate is a common medication indicated for duodenal ulcers and is used for gastric ulcers and gastritis. Off-label use of topical sucralfate has been reported for perineal and peristomal irritation to induce epithelial wound healing (5,6). We present a case report of a medically complex patient with a large stomal defect where topical sucralfate was used to create a smaller opening and allow for new G-tube device through the same stomal opening with minimal healing time without surgery.

## CASE REPORT

The patient was a 10-year-old female with a history of cerebral palsy, epilepsy, spastic quadriplegia with baclofen pump placement and revision, scoliosis with surgically inserted spinal growth rods, multiple orthopedic procedures for tendon release/lengthening, arthrodesis, tendon lengthening, and osteotomy. She was nonverbal with oral aversion and dysphagia since infancy. At 2 years of age, she underwent a 14F percutaneous endoscopic gastrostomy tube placement for dysphagia and oral aversion, and subsequent Mickey button 1.5 cm 14F changeout 6 months later.

**FIGURE 1.**
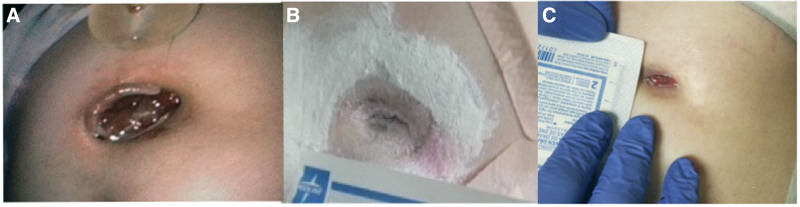
Photographic depiction of pre- and post-topical sucralfate application to G-tube stoma site. A) 2.0 x 1.5 cm defect at presentation. B) Reduction in size of defect 5 days later. C) Further reduction of ostomy site at 4 weeks after intial presentation. G-tube = gastrostomy tube.

Over the year prior to presentation, her G-tube tract slowly widened, to the point where the G-tube balloon easily fell out of her gastrocutaneous fistula. Family also reported irritation and bleeding around the G-tube site that soaked through 1–2 large gauze pads. She was seen in gastroenterology clinic and sent to the hospital for a direct admission. Endoscopy demonstrated gastritis, no false tracts, no gastric outlet obstruction, and a 2 cm × 1.5 cm defect at the dilated gastrostomy tract with prolapsed gastric mucosa (Fig. 1A). Medications included zonisamide 175 milligrams (mg) once per day, valproic acid 250 mg every day, diazepam 3 mg every 12 hours, glycopyrrolate 0.2 mg once a day, lansoprazole 0.5 mg/kilogram per day, ipratropium 0.02% nebulizer solution every day, and as needed medications including baclofen 10 mg tablet, albuterol nebulizer solution every 4 hours, diazepam rectal kit, and polyethylene glycol 8.5 gram every day.

Due to her complex medical history and anatomy, conservative management was initiated with the goal of reduction in gastrostomy site defect in an effort to utilize the same tract for replacement of the G-tube in a narrow gastric window. We used sucralfate 1 gram tablet, ½ tablet crushed sprinkled around the ostomy site 3 times a day. Powder was used to fill in the defect and any residual powder medication was gently cleaned off before the next application. A nasojejunal feeding tube was placed for feeding to allow healing of the gastric ostomy site. She tolerated the application and after 5 days showed reduction in the size of the tract (Fig. 1B). She was then discharged home with 12F Mickey button placed in the tract. She was seen by gastroenterology at 2 and 4 weeks after discharge, and the defect had reduced further in size without evidence of prolapsed gastric mucosa (Fig. 1C). A replacement G-tube was inserted about 1 month from discharge, and patient continued to clinically do well without significant G-tube complications.

## DISCUSSION

**FIGURE 2.**
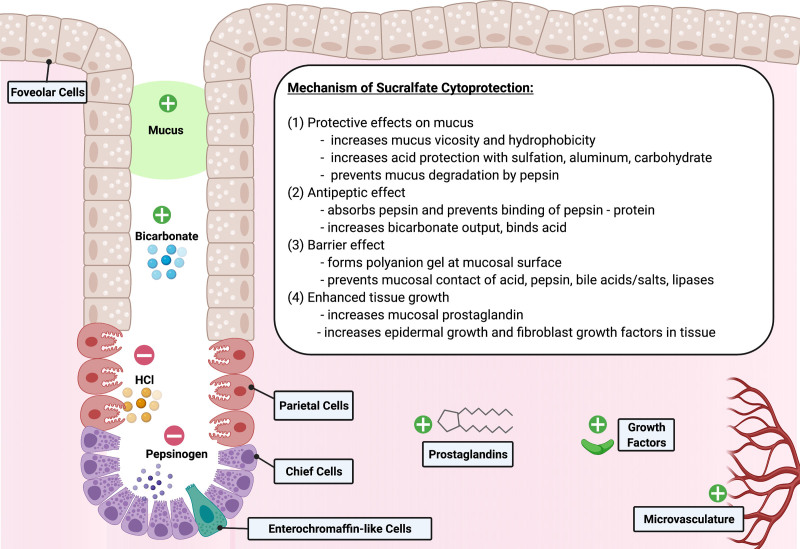
Mechanism of cytoprotection of sucralfate at gastric mucosa. Created with BioRender.com. HCl = hydrochloric acid.

Sucralfate is a poorly soluble complex salt composed of sucrose sulfate and aluminum hydroxide with the molecular structure of alpha-D-glucopyranoside, beta-D-fructofuranosyl-, octakis, aluminum (7). The mechanism of gastric cytoprotection is multifactorial (Fig. 2) (6,7). When placed in the presence of acid, the molecule releases positively charged aluminum and acquires a strong negative charge, thereby attracting positively charged chemicals such as proteins, peptides, and large mucin molecules (glycoproteins, glycolipoproteins) found at the gastric mucosa, which impair activation of peptic enzymes, acid, and bile salts/acids (7). The complex molecule chemically degrades to its aluminum salt and sucrose sulfate components that form a gel-like substance increasing the viscosity and hydrophobicity of ambient mucus (7). Sucralfate demonstrates binding to both epidermal growth factors and fibroblast growth factors, which accommodate granulation tissue through angiogenesis, epithelialization, and deposition of extracellular matrix (6,7). Prostaglandin production is also thought to be induced by sucralfate leading to endothelial proliferation and gastric epithelial homeostasis (6,7). In culmination, growth factors and prostaglandin induce increased vascular blood flow and facilitate wound healing.

Application of topical sucralfate with placement of a post-pyloric feeding tube as described in this case report represents conservative management of G-tube site size reduction for pediatric gastroenterologists. Although it is tempting to place a larger G-tube into a widened stoma site, this may worsen the underlying problem of the expanded stoma site. Application of sucralfate in G-tube site size reduction, closure, and reuse demonstrates a new role for this medication for pediatric gastroenterologists and may save patients from unnecessary procedures and surgeries and shorter healing time. This is also a technique and application that can be readily taught to caregivers at home, rather than seeking medical intervention.

## ACKNOWLEDGMENTS

The authors would like to thank the patient’s parents/guardians who provided informed consent for publication of this case report.

## References

[1] Khalil ST Uhing MR Duesing L Visotcky A Tarima S Hang Nghiem-Rao T. Outcomes of infants with home tube feeding: comparing nasogastric versus gastrotomy tubes. J Parenter Enteral Nutr. 2017; 41:1380–1385. 10.1177/0148607116670621 PMC5727911 27647478

[2] Janik TA Hendrickson RJ Janik JS Landholm AE. Analysis of factors affecting the spontaneous closure of a gastrocutaneou fistula. J Peds Surg. 2004; 39:1197–1199. 10.1016/j.jpedsurg.2004.04.007 15300526

[3] Wyrick DL Bozeman AP Smith SD. Persistent gastrocutaneous fistula: factors affecting the need for closure. J Pediatr Surg. 2013; 48:2506–2510. 24314194 10.1016/j.jpedsurg.2013.06.001

[4] Alshafei A Deacy D Antao B. Risk factors for a persistent gastrocutaneous fistula following gastrostomy device removal: a tertiary center experience. J Indian Assoc Pediatr Surg. 2017; 22:220–225. 28974874 10.4103/jiaps.JIAPS_205_16 PMC5615896

[5] Hayashi AH Lau HY Gillis DA. Topical sucralfate: effective therapy for the management of resistant peristomal and perineal excoriation. J Pediatr Surg. 1991; 26:1279–1281. 1812256 10.1016/0022-3468(91)90598-n

[6] Masuelli L Tumino G Turriziani M Modesti A Bei R. Topical use of sulcralfate in epithelial wound healing: clinical evidence and molecular mechanisms of action. Recent Pat Inflamm Allergy Drug Discov. 2010; 4:25–36. 19832693 10.2174/187221310789895649

[7] McCarhy DM. Sucralfate. N Engl J Med. 1991; 325:1017–1025. 1886624 10.1056/NEJM199110033251407

